# Antimicrobial and Antioxidant Properties of a Bacterial Endophyte, *Methylobacterium radiotolerans* MAMP 4754, Isolated from *Combretum erythrophyllum* Seeds

**DOI:** 10.1155/2020/9483670

**Published:** 2020-02-18

**Authors:** Mampolelo M. Photolo, Vuyo Mavumengwana, Lungile Sitole, Matsobane G. Tlou

**Affiliations:** ^1^Department of Biochemistry, Faculty of Science, University of Johannesburg, Auckland Park Campus, Johannesburg, South Africa; ^2^DST-NRF Centre of Excellence for Biomedical Tuberculosis Research, South African Medical Research Council Centre for Tuberculosis Research, Division of Molecular Biology and Human Genetics, Faculty of Medicine and Health Sciences, Stellenbosch University, Tygerberg Campus, Cape Town, South Africa; ^3^Department of Biochemistry, School of Physical and Chemical Sciences, Faculty of Natural and Agricultural Sciences, North-West University, Mafikeng Campus, South Africa

## Abstract

This study reports on the isolation and identification of *Methylobacterium radiotolerans* MAMP 4754 from the seeds of the medicinal plant, *Combretum erythrophyllum,* for the purposes of investigating antimicrobial and antioxidant activities from this endophyte. The strain identity was confirmed by 16S rRNA-based phylogeny and Scanning Electron Microscopy (SEM). Ethyl acetate and chloroform (1 : 1 v/v) extracts from the endophyte were tested for antimicrobial and antioxidant activity on a total of 7 bacterial species (3 Gram-positive and 4 Gram-negative) using the standard Minimum Inhibitory Concentration (MIC) protocol and Quantitative Radical Scavenging activity using the 2, 2-diphenyl-1-picrylhydrazyl (DPPH) assay, respectively. The MICs were recorded at 250 *μ*g/mL for *B. subtilis* ATCC 19659, *B. cereus* ATCC 1076, *E. coli* ATCC1053, and 62.5 *μ*g/mL for *K. oxytoca* ATCC 13182 and *M. smegmatis* ATCC 21293, while an IC_50_ of 5.65 *μ*g/mL was recorded with the DPPH assay. Qualitative phytochemical analysis was positive for alkaloids, flavonoids, and steroids. Gas chromatography/mass spectrometry (GC/MS) analysis revealed the presence of 9-octadecene, 2,4-dinitrophenyl acetate, and 2(5H)-furanone, which have been previously reported for the targeted activities. *M. radiotolerans* MAMP 4754 tested positive for antimicrobial and antioxidant activity and this is linked to the production of plant-derived secondary metabolites by this strain.

## 1. Introduction

The exponential increase in the number of drug-resistant pathogens coupled with immune-suppressing diseases has rendered infectious disease control a major global challenge. This challenge necessitates the search for new bioactive compounds with pharmaceutical potential [1–3]. Over the years, continued bioprospecting of medicinal plants has generated 47% of drugs currently approved by the United States of America's Food and Drug Administration (FDA), with only 3% of these being antimicrobial [4]. The pharmaceutical properties of medicinal plants have been linked to the production of a wide variety of structurally diverse phytochemicals which include alkaloids, flavonoids, terpenes, steroids, curcumins, saponins, and phenolics, all of which can potentially serve as drug lead candidates for the development of antimicrobials and resistance modifiers [5, 6].

According to the International Union for Conservation of Nature and the World Wildlife, there are an estimated 80 000 recognized medicinal flowering plant species globally, 15 000 of which are being threatened with extinction due to habitat destruction and overharvesting caused by unregulated informal markets [7, 8]. In order to conserve currently endangered medicinal plant species from permanent disappearance, there is an increased interest in systems which offer potentially bioactive and chemically diverse compounds like those found in plants but with negligible environmental effects.

Endophytes are endosymbiotic microorganisms (commonly bacteria or fungi) that systematically colonize and proliferate within plant tissues without causing any signs of disease or harm [9]. In colonizing plant tissue, endophytes are also capable of establishing a symbiotic relationship with the plant thus making them efficient biocontrol and medicinal agents. Several research reports have demonstrated the activity of bacterial endophytes against various pathogens [10, 11]. As such, there is continued research interest in developing drugs from endophytic compounds which could serve as an alternative to synthetic pharmaceuticals and/or plant-derived medicines. Endophytes are known to promote plant growth, enhance defence, increase abiotic and biotic stress tolerance, and improve nutrient acquisition [12]. Endophytes may actively modulate the host's biosynthesis pathways and gene expression systems to increase the production of significant secondary metabolites. An interesting case being that of the medicinal plant *Withania somnifera* whereby some isolated endophytes could induce the production of withaferin A (abundantly produced in the leaves) in the roots and while some upregulate the expression of 1-deoxy-D-xylulose-5-phosphate synthase (DXS) and 1-deoxy-D-xylulose-5-phosphate reductase; (DXR) genes [13].

A key advantage of endophytes is that they can be easily isolated and cultured and are amenable to genetic manipulations and can be scaled up for bioactive compound production [14]. Considering the importance of bacterial endophytes to both plant and human health, there is an increased focus on developing endophytes into herbal remedies. The current study is based on *Methylobacterium radiotolerans* MAMP 4754 [15], a bacterial endophyte isolated from the seeds of *Combretum erythrophyllum.* The *Methylobacterium genus* is composed of bacterial species that are Gram-negative, pink pigmented [16], rod-shaped, strictly aerobic, and facultative methylotrophs [17]. Members of this *genus* are commonly found in various environments due to their phenotypic plasticity [18, 19]. Crude and partially purified extracts of the *Methylobacterium genus* have been shown to possess antimicrobial, anticancer, and antioxidant properties [20, 21]. The purpose of this study was to, therefore, analyse the antimicrobial and antioxidant activity of *Methylobacterium radiotolerans* MAMP 4754. Thus, results obtained from this study will further affirm the significance of the *Methylobacterium* genus as a source of pharmaceutically relevant bioactivity.

## 2. Method and Materials

### 2.1. Plant Sample Collection and Identification

Healthy disease-free *C. erythrophyllum* with its dry fruits was harvested from Mukula village situated east of Thohoyandou (22.8600 S 30.5661 E), Limpopo province, South Africa. The fruits were collected on-site and transported at 4°C in sterile polyethylene bags. The identification of the plant was done at the University of Johannesburg Herbarium (JRAU) by Professor Annah Moteetee. The sample specimen was assigned Photolo-Mavumengwana-2015 and species name *C. erythrophyllum.*

### 2.2. Isolation of Bacterial Endophytes

Bacterial endophytes were isolated from the seeds of the plant by a method described by Jasim et al. [22]. Briefly, surface sterilization of the fruits was done by firstly washing them with tap water to remove dust, followed by treatment with Tween 80 for 10 minutes with vigorous shaking, and then rinsing with sterile distilled water. The washing was continued with 70% ethanol and 1% sodium hypochlorite (NaOCl) for 1 and 10 min, respectively, followed by thorough rinsing with sterile distilled water three times. The final wash was spread on Luria-Bertani (LB) agar plates to determine the success of the surface sterilization process. The outer surface of sterile fruits was then trimmed off to access the delicate seeds which were then macerated in phosphate buffer saline (PBS) for bacterial endophyte isolation. Serial dilutions of up to 10^−3^ were prepared and 0.1 mL of the dilutions was spread on LB agar plates in triplicates. These plates and the controls were incubated at 30°C for up to 3 days with daily monitoring for the occurrence of colonies. The colonies that emerged after the incubation period were subcultured several times on LB agar to obtain pure cultures that were then stored in 50% glycerol at −80°C for long-term future use.

### 2.3. DNA Extraction and 16S rRNA Gene Amplification

A frozen stock of one of the isolates (*M. radiotolerans* MAMP 4754) was revived by spreading inoculum on LB agar and incubating overnight at 30°C. A single colony was picked to inoculate LB broth which was incubated until an optical density measured at 600 nm (OD_600_) was about 0.4-0.5. Genomic DNA was then extracted from the culture using DNeasy blood and tissue kit (Qiagen, Germany) following the manufacture's protocol. The extracted genomic DNA was then quantified using a NanoDrop™ ND-2000 UV-vis spectrophotometer (Thermo Fisher Scientific, USA).

### 2.4. Polymerase Chain Reaction (PCR) Amplification and Sequencing of the 16S rRNA Gene

The 16S rRNA gene of the bacterial endophyte was amplified according to a method described by Tsuchida et al. [23]. The 16S rRNA gene of the endophyte was then amplified using the universal primers BacID 1F (5′-AGAGTTTGATCTGGCTCAG-3′) and BacID 1500R (5′-AAGGAGGTGWTCCARCC-3′) which were bought from Inqaba Biotechnological Industries (Pretoria, South Africa). Polymerase chain reaction (PCR) was in 50 *μ*L total volumes using the following conditions; 1X initiation cycle at 92°C for 2 min, 30X denaturation cycles at 92°C for 30 sec, 30X primer annealing cycles at 52°C for 30 sec, 30X extension cycles at 72°C for 2 min, and a 1X elongation cycle at 72°C for 2 min followed by termination at 4°C. The PCR products were analysed on a 1.5% agarose gel by electrophoresis at a constant 100 V and 200 mA. The positive products were then excised from the gel and purified using the GeneJet gel extraction kit (Thermo Fisher Scientific, USA). The purified PCR products sequenced at Inqaba Biotechnical Industries.

### 2.5. Phylogenetic Analysis

The individual sequences were assembled within Geneious (Geneious 8.1.9) and a contig was formed. This was preceded by a BLAST search on the NCBI GenBank nucleotide sequence database (https://www.ncbi.nlm.nih.gov/genbank/). Sequences of species closely matching the BLAST query sample along with their closely related taxa were obtained for the phylogenetic analysis. A matrix was generated with the query samples and the closest matching sequences, which were then aligned using Geneious Alignment at a Cost Matrix of 70% similarity. Using a Heuristic search followed by a Bootstrap analysis, a parsimonious phylogenetic tree was constructed using Parsimony (PAUP ∗ 4.0).

### 2.6. Morphological Characterization of *M. radiotolerans* MAMP 4754

To establish the morphological characteristics such as shape and Gram stain reaction, pure colonies were analysed by methods described by Collins et al. [24]. The slides were viewed using a compound bright-field microscope (OLYMPUS CH20BIMF200) with 100x magnification [25]. Morphological characteristics such as shape and Gram stain reaction and purity of stock cultures were analysed using a previously reported method. The prepared slides were viewed using a compound bright-field microscope (OLYMPUS CH20BIMF200) at 100x magnification.

### 2.7. Scanning Electron Microscopy

Scanning electron microscopy (SEM) was performed using methods described by Golding et al. [26]; and Schadler et al. [27]. In brief, *M. radiotolerans* MAMP 4754 was grown at 30°C, shaking at 150 rpm, in LB broth (NB) until an OD_600_ was 0.4–0.8. The bacterial cultures were then centrifuged at 10 823 rpm for 10 min and the supernatant discarded. Cells were then washed with sterile distilled water and the pellet was fixed with (1 : 1v/v) of 1% formaldehyde and 2% glutaraldehyde for 1 h at room temperature (25°C). Following fixation, samples were centrifuged at 10 823 rpm for 10 min, followed by discarding of the supernatant and washing of the pellet with 1 mL of sterile distilled water. For dehydration, bacterial cells were treated with increasing concentrations of ethanol (30, 50, 70, 90, 95, and 100%) with 10 min intervals. Samples were stored open at 4°C overnight and mounted on SEM stubs the following day. These were then coated with gold using Emscope SC 500 and viewed using Tescan Vega 3 (Tescan-Orsay, Czech Republic), which was operated at 6 kV accelerating voltage.

### 2.8. Extraction of *M. radiotolerans* MAMP 4754 Secondary Metabolites

The extraction of secondary metabolites from *M. radiotolerans* MAMP 4754 was carried out using the method previously described in Balachandran et al. [20] with minor modifications. Briefly, LB broth (1 L) was prepared in 2 L Erlenmeyer flasks and autoclaved at 121°C for 20 min. Each of the 2 L flasks was inoculated with the endophytic bacteria and incubated at 28°C for 7 days shaking at 200 rpm. After the 7^th^ day, the culture was centrifuged at 10 000 rpm for 15 minutes for biomass removal. Equal volumes of ethyl acetate and chloroform (1 : 1 v/v) were added to the supernatant followed by vigorous shaking. The organic solvent layer was collected in a conical flask and the organic layer was concentrated using a vacuum rotary evaporator at 40°C. The extract was transferred to a 5 mL sterile vial and left to dry at room temperature. The phytochemical screening of *M. radiotolerans* MAMP 4754 crude extract was adopted from [28, 29] with minor modifications shown in Table 1.

### 2.9. Antimicrobial Activity of Crude Extract from *M. radiotolerans* MAMP 4754

Evaluation of the antimicrobial activity of the secondary metabolite crude extract was carried out using the disc diffusion method as previously described by Hoelzer et al. [30] and Zhang et al. [31]. Seven pathogenic bacterial strains (Gram-positive strains: *Bacillus cereus* ATCC 1076, *Bacillus subtilis* ATCC 19659, *Mycobacterium smegmatis* ATCC 21293, *Enterococcus faecalis*, Gram-negative strains: *Escherichia coli* ATCC 10536, *Pseudomonas aeruginosa* ATCC 25922, *Klebsiella oxytoca* ATCC 13182) and a fungal strain *Candida albicans* were grown overnight at 37°C in Mueller-Hinton (MH) broth, which was adjusted to McFarland Standard No. 0.5 such that the concentration was 10^7^ to 10^8^ colony-forming units per millilitre (CFU/mL). The pathogenic strains were further spread on MH agar plates and sterilized circular paper discs (4 mm), soaked with the bacterial endophyte crude extract were placed on the plates containing a lawn of bacterial culture of the test pathogenic strains. For the positive control, 10 *μ*L of 1 mg/mL Ampicillin, Kanamycin, and Ketoconazole was also impregnated on the paper discs as described above and for the negative control, respective solvents (chloroform and ethyl acetate) were utilized. All the plates were then incubated at 37°C for 48 to 72 hours and antimicrobial activity was assessed by observing and measuring the zone of inhibition in mm. The antimicrobial experiments were performed in triplicate (*n* = 3).

### 2.10. Determination of Minimum Inhibitory Concentration

The Minimum Inhibitory Concentration (MIC) was used in the study to determine the antimicrobial activity of *M. radiotolerans* MAMP 4754 by serial microdilution in a 96-well microplate [32, 33]. The same pathogenic strains that were utilized in the disc diffusion assay were used for the MIC test. The bacterial strains were grown at 37°C for 12 to 24 hours (depending on the growth rate of each pathogenic strain) in MH broth. To the wells in column A of the microtiter plate, a 100 *μ*L of MH broth and 100 *μ*L of *M. radiotolerans* MAMP 4754 extract (1 mg/mL) was added and mixed. Columns B to H were also loaded with 100 *μ*L of MH broth and serial dilutions were carried out from column A to a final concentration of 3.9 *μ*g/mL in column H. This was done in triplicate. Following the dilution process, each well was loaded with 100 *μ*L of bacterial culture to a 0.5 McFarland's standard. Similarly, this was done with the positive controls of Ampicillin, Kanamycin, and Ketoconazole and for the negative control, 0.1% DMSO was used. The microtiter plate was sealed with parafilm and incubated at 37°C for 24 hours. After incubation, 10 *μ*L of 4 mg/mL iodonitrotetrazolium chloride was added to each well. MIC was recorded as the lowest concentration with clear wells, which indicated the absence of microbial growth.

### 2.11. Scavenging 2, 2-Diphenyl-1-picrylhydrazyl (DPPH) Free Radical Assay

Antioxidant activity of the *M. radiotolerans* MAMP 4754 crude extract was studied using the 2, 2-diphenyl-1-picrylhydrazyl (DPPH) free radical scavenging assay. This was carried out using a modified method previously described by Takao et al. [34], with minor modifications. The crude extract of *M. radiotolerans* MAMP 4754 was dissolved in ethanol and mixed with a 90 *μ*M DPPH ethanol solution to give a final concentration of 0.78–100 *μ*g/mL. Ethanol was used as solvent control, while ascorbic acid was used as a positive control. The extract with DPPH was incubated in the dark at room temperature for 30 minutes. The absorbances were measured at 492 nm (Bio-TekSynergy HT Multi-Detection Microplate Reader; USA). All experiments were done in triplicate and the IC_50_ was calculated graphically with percentage inhibition calculated as follows: %inhibition = 100 × ((absorbance of control − absorbance of the sample)/absorbance of control).

### 2.12. Gas Chromatography High-Resolution Time-of-Flight Mass Spectrometry (GC-HRTOFMS)

Secondary metabolites were identified using gas chromatography high-resolution time-of-flight mass spectrometry (GC-HRTOFMS (LECO Corporation St. Joseph, MI, USA)), operating in high-resolution, equipped with a Gerstel MPS multipurpose autosampler (Gerstel Inc. Germany). For the analysis, the samples were run in a 30 m × 0.25 mm capillary column with a film thickness of 0.25 *μ*m. The carrier gas was helium and it was maintained at a column flow rate of 1 mL/min. A 1 *μ*L sample of the extract was injected and the column temperature was maintained at 75°C followed by temperature programming at 10°C/min to 235°C for 2 mins, and finally to 300°C at a rate of 40°C/min for 3 min (Scan range: 45–500 *m*/*z*). The mass spectrometer and transfer line were held at 250°C. Peak picking, peak and retention time alignment, and detection and matching were done on ChromaTOF-HRT® software (LECO Corporation, St Joseph, MI, USA). A signal to noise (S/N) ratio of 100 was used and similarity/probability match was ˃70% before a name was assigned to a compound using Mainlib, NIST and Feihn metabolomics database through comparison of the mass spectra data, molecular formula, and the retention time.

### 2.13. Statistical Analysis

The resulting data were analysed using analysis of variance (ANOVA). The antimicrobial and DPPH data were described as mean ± standard deviations (SD). This analysis was done using Microsoft Excel Office 365 ANOVA. *p* values less than 0.05 were considered to be statistically different.

## 3. Results and Discussion

### 3.1. Molecular and Morphological Identification of *M. radiotolerans* MAMP 4754

**Figure 1:**
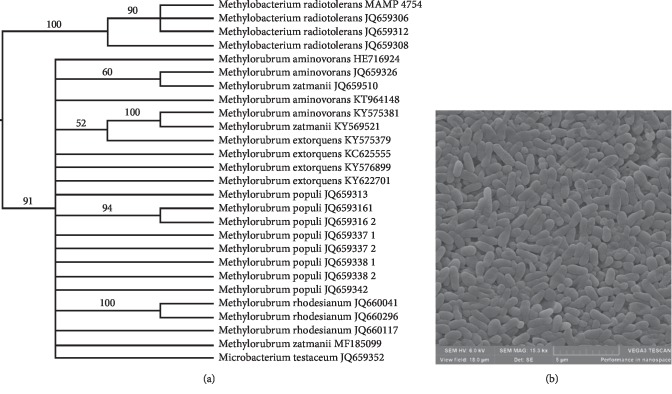
(a) Phylogenetic tree derived from 16S rRNA gene sequences showing the relationship between endophyte *M. radiotolerans* MAMP 4754 and species belonging to the genus *Methylobacterium.* Bootstrap values were expressed as percentages of 100 replications. (b) Scanning electron micrograph showing the rod-shaped morphology of *M. radiotolerans* MAMP 4754 cells.

Prior to endophyte isolation, *C. erythrophyllum* seeds were surface sterilized and the sterilization method was adequate as none of the control plates showed any microbial growth. The 16S rRNA gene of the bacterial endophyte was amplified and the expected amplicon size of 1500 bp was obtained (data not shown). The sequencing of the amplicon and similarity/BLAST searches revealed that the bacterial isolate, *M. radiotolerans* MAMP 4754, shared 99% homology to bacterial species belonging to *Methylobacterium* genus as indicated in Figure 1(a). The genus has over fifty published species; however, based on the 16S rRNA gene, multilocus sequence analysis, and genomic and phenotypic data, fifty-two *Methylobacterium* species can no longer be regarded in this genus. A new genus, *Methylorubrum* gen. nov. has been proposed to accommodate 11 species, which were previously held in the *Methylobacterium* genus [35]. Additionally, this isolate is in a *Methylobacterium* clade that is sister to *Methylorubrum gen nov* clade, as demonstrated in Figure 1(a).

The Gram stain reaction indicated that *M. radiotolerans* MAMP 4754 is a rod-shaped, Gram-negative bacterium as it did not retain the crystal violet stain used in the Gram staining method (data not shown) [36, 37]. The bacterial morphology (with uniform cells indicating that the bacterial cultures were pure) was further confirmed by the SEM electron micrograph (Figure 1(b)).

The isolation of endophytic bacteria has been reported for several medicinal plants such as *Catharanthus roseus*, *Ocimum sanctum* and *Mentha arvensis* [38], *Lonicera japonica* [39], and *Ferula songarica* [40]. However, there are currently a few reports on the isolation and characterization of endophytic *Methylobacterium* and in the context of bioactivity, some of the reports confirmed the antimicrobial compounds [41, 42] and production of defensin-like antimicrobial peptides [43].

### 3.2. Phytochemical Analysis

The results of the phytochemical screening indicated that *M. radiotolerans* MAMP 4754 consists of alkaloids, flavonoids, and steroids and contained no tannins and saponins (Table 1). This observation indicates that the bacterium shares some common secondary metabolite biosynthetic pathways with the host plant since *Combretum* species are rich in intermediate polar compounds such as flavonoids, stilbenoids, and triterpenoids [44–46]. Flavonoids are polyphenolic compounds generally found in plants, vegetables, fruits, tea, and coffee [47] and they are known to show antimicrobial, anticancer, anti-inflammatory, antiallergic, antioxidant, and antiviral properties [48]. Alkaloids also have pharmacological, veterinary, and medical properties [49]. Various alkaloids used in anticancer drugs include Camptothecin and Vinblastine, while Morphine and Codeine are used as analgesics and ephedrine is used for relieving asthma [50].

Bioactive compound production by endophytes is related to the evolution of the host microorganisms, which may have incorporated genetic information from higher plants through horizontal gene transfer [51–53]. This enables host plant adaptation and the achievement of functions which are vital to the plant such as protection from pathogens, insects, and grazing animals [52, 54]. Therefore, the presence of flavonoids, alkaloids, and steroids in *M. radiotolerans* MAMP 4754 is indicative of the therapeutic potential that endophytes contract from their hosts.

### 3.3. Antimicrobial Activity and MIC

Crude secondary metabolites from *M. radiotolerans* MAMP 4754 were extracted using ethyl acetate and chloroform and the MICs for each pathogen tested are shown in Table 1. The ethyl acetate and chloroform extract (1 mg/mL) showed antibacterial and antifungal activity against selected pathogenic strains (Table 2). The results obtained indicated a significant difference in the antimicrobial activity of *M. radiotolerans* MAMP 4754 crude extract against all pathogens compared to the antibiotic controls.

*M. radiotolerans* MAMP 4754 was extracted using ethyl acetate and chloroform solvents. The ethyl acetate and chloroform extract (1 mg/mL) showed antibacterial and antifungal activity against selected pathogenic strains (Table 2). Statistical analysis of the antimicrobial activities showed that the*p* value was less than 0.05, which indicates that there was a significant difference in the antimicrobial activity of *M. radiotolerans* MAMP 4754 against all pathogens compared to the control antibiotics. The crude extract, resuspended in ethyl acetate, showed zones of inhibition ranging from 12 to 24 mm with the most significant inhibition observed on *K. oxytoca*, *B. cereus*, and *M. smegmatis* at 24, 20, and 18 mm, respectively ([Supplementary-material supplementary-material-1]; supplementary data). The extract resuspended in chloroform showed zones of inhibition ranging from 12 to 17 mm with the most significant inhibition observed on *B. subtilis* ([Supplementary-material supplementary-material-1]; supplementary data). These results were in correlation to previous studies conducted by Pansanit and Pripdeevech [55] and Salehan et al. [56], where ethyl acetate extracts of endophytes from *Zingiber cassumunar* proved to be the most effective inhibitors of fungal growth, Gram-positive and negative bacteria [55, 56]. This is also consistent with the various literature reports, showing that ethyl ether and ethyl acetate, are hydrogen bond acceptor molecules and can, therefore, extract electron donor solutes better than chloroform [57]. Furthermore, in their study Kavitha et al. [58] effectively extracted antimicrobial compounds from *Streptomyces* sp. TK-VL_333 using ethyl acetate [58]. This was further confirmed in a study by Mtunzi et al. [59] where ethyl acetate fractions were observed to have been the best extractant for antimicrobial compounds in an MIC assay. Taken together, the antimicrobial activity observed in the current study along with previous studies can be associated with the presence of phenolic and polyphenolic compounds [60, 61]. This, therefore, suggests that bacterial endophytes can produce similar bioactive compounds as their host plant [62–64].

The MIC values of the secondary metabolites ranged from 62.5 *μ*g/mL to 250 *μ*g/mL. The highest value was observed with *B. subtilis*, *B. cereus*, and *E. coli* with an MIC value of 250 *μ*g/mL (Table 2). A notable value of 62.5 *μ*g/mL was observed on *K. oxytoca* and *M. smegmatis*, showing that *M. radiotolerans* MAMP 4754 has great potential for the development of compounds containing bioactivities against human pathogenic microorganisms as shown in Table 2. Crude extracts exhibiting activity at a concentration of 1000 *μ*g/mL or lower are considered significantly active [65]. Interestingly, in a study of antimicrobial activity of *C. erythrophyllum* plant crude extracts, MIC values ranging from 80 *μ*g/mL to 125 *μ*g/mL were observed, which compares to the findings of the current study. Furthermore, the results that were obtained for the potential activity of the *M. radiotolerans* MAMP 4754 crude extract against the tested pathogens correlate with those of Martini and Eloff [66] on *Combretum* spp. with the lowest value of 50 *μ*g/mL [66]. This activity can be related to the presence of the flavonoid and phenolic content (Table 3) [78, 79]. This is an initial report on the antimicrobial activity of secondary metabolites from an endophyte isolated from *C. erythrophyllum*.

### 3.4. Antioxidant Activity

**Figure 2:**
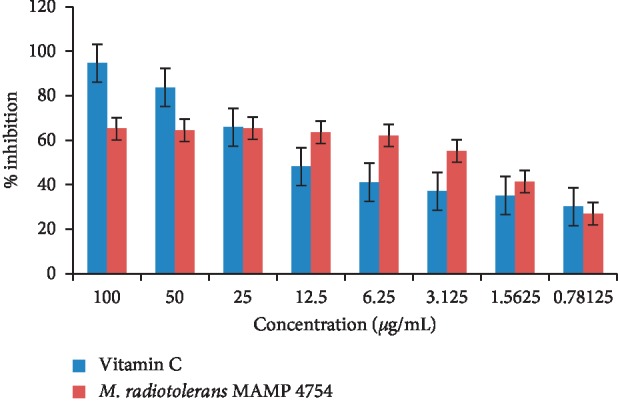
DPPH free radical scavenging activity of *M. radiotolerans* MAMP 4754 crude extract (*n* = 3). Ascorbic acid (vitamin C) was used as a positive control. IC_50_ values were 6.69 *μ*g/mL and 5.65 *μ*g/mL for the ascorbic acid and the crude extract, respectively. Statistical analysis was performed using ANOVA and the differences were considered significant at *p* < 0.05.

DPPH free radical assay was used in this study to test the scavenging effects of the *M. radiotolerans* MAMP 4754 extract. DPPH assay is a commonly used tool for the evaluation of the scavenging effect of natural products based on the quenching of stable coloured radicals [80]. However, DPPH is a preliminary method for antioxidant activity and should, therefore, be verified by other more sensitive assays such as the ferric reducing antioxidant power (FRAP) test and *β*-carotene bleaching assay [81, 82]. Ascorbic acid, which had an IC_50_ of 6.69 *μ*g/mL, was used as a standard control because of the characteristic antioxidant and radical scavenger activities (Figure 2). The *M. radiotolerans* MAMP 4754 extract showed antioxidant property at IC_50,_ of 5.65 *μ*g/mL. A low IC_50_ value is indicative of greater radical scavenging activity [83]. Therefore, the IC_50_ of *M. radiotolerans* MAMP 4754 observed in this study suggests that the extract of *M. radiotolerans* MAMP 4754 is an effective radical scavenger and can be used as a potential antioxidant supplement. These findings correspond to a previous study of a *C. erythrophyllum* ethyl acetate plant extract showing a free radical scavenging activity at IC_50_ 4.3 *μ*g/mL [59]. In their study Mtunzi et al. [59] ranked the activity of the plant crude extracts in the following order: ethyl acetate > dichloromethane > water > acetone > hexane [59]. Interestingly, high antioxidant activity in DPPH assays is associated with phenolic compound quantity in crude extracts [80, 84, 85] and this can be seen in our GC/MS findings (Table 3) where several phenolic compounds were detected and identified.

Phenolic compounds were previously said to possess ideal structural chemistry for free radical scavenging activity [86]. Furthermore, this activity corresponds to the presence of flavonoid compounds, which were detected in the phytochemical analysis (Table 1). Flavonoids have also been reported to play an important role in the reduction of lipid peroxidation and thus act as primary and secondary antioxidants [87]. Total flavonoid content has also been attributed to free radical scavenging activity in fungal endophyte extracts of *Fritillaria uni bracteate* var. *wabuensis* [88].

The free radical scavenging effects of the crude extract showed significant antioxidant potentials in comparison to the ascorbic acid, with this being a great indication that the endophytes can indeed be a good source of bioactive compounds relevant in the development of novel therapeutic drugs.

### 3.5. Gas Chromatography High-Resolution Time-of-Flight Mass Spectrometry Analysis

The use of endophytes as a source of bioactive compounds is an advantageous alternative as they are known to produce similar secondary metabolites as their host plant. *C. erythrophyllum* has several medicinal properties such as antibacterial [63, 64], antifungal [62], anti-inflammatory/oxidizing [89, 90] and antitumor [91], and it continues to be used as a traditional medicinal plant. In order to identify the compounds responsible for the observed antimicrobial and antioxidant activities (Table 3) GC-HRTOFMS was carried out. The GC-HRTOFMS analysis of volatile components in the extract of *M. radiotolerans* MAMP 4754 indicated the presence of a class of fatty acids (9-octadecene, 3-eicosene, 11-tricosene, hexadecane) and several phenolic compounds all of which are linked to the notable antimicrobial and antioxidant properties of *M. radiotolerans* MAMP 4754 observed (Table 3). These findings are similar to those of Yogeswari et al. [92] and Naragani et al. [93] who, from the fungus *Monochaetia kansensis,* also observed antimicrobial activity owing to the presence of 9-octadecene, 3-eicosene, and 11-tricosene [92, 93]. In addition, these fatty acids are found in abundance in several plants and have also been reported to have anticancer and antioxidant activity [67, 68, 94].

Antioxidant activity of phenolic compounds, identified herein, can be described in terms of their intrinsic bioactivity as free radical scavengers or indirectly as modulators of intracellular pro- and antioxidant enzymes [95]. Phenolic compounds have been shown to exert antioxidant properties both *in vitro* and *in vivo* [96]. One of the phenolic compounds identified in this study was p-tert-butyl (Table 3). This observation corresponds to that of Balasundram et al. [97] who reported on the antifungal, anticancer, and antioxidant activity of p-tert-butyl isolated and purified from *Lactococcus* sp. [97].

Furan derivatives, which were also detected in the current study, are used in the cosmetics and pharmaceutical industry due to their medicinal properties [71–73]. In a previous study by Zekeya et al. [73] and Sharma et al. [72], 2furanmethanol 5-methyl was detected from a methanol : chloroform extract of *Gunnera perpensa.* In that study, 2-furanmethanol 5-methyl was detected with a percentage area of 0.34% and was reported to possess bioactivities such as anticancer, anti-inflammatory, and antimicrobial activity [72, 73]. Other important bioactive compounds detected in this study were siloxane derivatives which have been shown to possess antifungal and antibacterial activity [75]. Antioxidant capacity has also been reported in methanol extracts of *Merremia aegyptia* and *Merremia dissecta* [77]. In their study, Joshi et al. [77] detected the presence of siloxane derivatives such as 3-ethoxy-1,1,1,7,7,7-hexamethyl-3,5,5-tris (trimethylsiloxy) tetrasiloxane in the methanol extracts using GC-MS analysis [77]. Results obtained from our study are similar and comparable to those from previous reports and therefore justify the possible development of *M. radiotolerans* MAMP 4754 for pharmacological action. The data presented herein further confirms the notion that endophytes are a great source of bioactive compounds that can be utilized in drug discovery and could be a great source of natural antioxidants. Further studies are required to provide a better understanding of endophytes and the secondary metabolites produced and possibly elucidating the mechanism of action.

## 4. Conclusion

To our knowledge, this is the first study to report on the antimicrobial and antioxidant bioactivities of *M. radiotolerans* MAMP 4754. The ethyl acetate extract of *M. radiotolerans* MAMP 4754 exhibited a higher degree of antibacterial and antifungal activity showing high zones of inhibition against seven human microbial pathogens compared to the chloroform extract. Furthermore, the *M. radiotolerans* MAMP 4754 crude extract showed low MIC values of 62.5 *μ*g/mL against *K. oxytoca* and *M. smegmatis*. This is indicative of a high antimicrobial potential of the endophyte. Additionally, *M. radiotolerans* MAMP 4754 extract showed high antioxidant activity. The phytochemical analysis of *M. radiotolerans* MAMP 4754 revealed the presence of flavonoids, steroids, and alkaloids. Moreover, the screening of bioactive compounds of the ethyl acetate extract of *M. radiotolerans* MAMP 4754, indicated the presence of different compounds that have been previously reported to show potential applications in pharmaceuticals and agriculture. As such, future studies of bacterial endophytes can lead to the development of novel therapeutic drugs from the analysis of the secondary metabolites they produce and could aid in understanding the biochemical pathways for synthesis of some of these biologically active compounds.

## Figures and Tables

**Table 1:** Phytochemical analyses for *M. radiotolerans* MAMP 4754 crude extract.

Phytochemical test	Method	Observations	*M. radiotolerans* MAMP 4754 crude
Tannins	Add 2-3 drops of FeCl_3_ to 1 mL endophyte extract	Blackish-blue or blackish-green colour	−
Alkaloids	Add 2-3 drops of Dragendorff's reagent to 1 mL endophyte extract	Turbidity or precipitation formation	+
Flavonoids	Add 2-3 drops of NaOH to 1 mL endophyte extract	Yellow colour formation	+
Saponins	Add 2-3 drops of olive oil to 5 mL endophyte extract. Shake vigorously	Froth or foam formation	−
Steroids	Add 1 mL of CHCl_3_. Add 2-3 drops of conc. H_2_SO_4_ to 1 mL endophyte extract.	Reddish brown ring	+

The (+) and (−) represent the presence and absence of the phytochemicals.

**Table 2:** Minimum inhibitory concentration of *M. radiotolerans* MAMP 4754 crude extract (*μ*g/mL). Kanamycin, Ampicillin, and Ketoconazole were used as positive controls (1 mg/mL). Data were reported as mean values of the crude extract tested in triplicate. Statistical analysis was performed using ANOVA and the differences were considered to be significant at *p* < 0.05.

Organism	Endophytic crude extract (*μ*g/mL)	Antibiotic control (*μ*g/mL)
^*∗*^ *Bacillus subtilis*	250	22
^*∗*^ *Bacillus cereus*	250	22
^∗^ *Klebsiella oxytoca*	62.5	20
^∗^ *Pseudomonas aeruginosa*	125	30
^∗^ *Mycobacterium smegmatis*	62.5	14
^∗^ *Enterococcus faecalis*	125	22
^∗^ *Escherichia coli*	250	14
^*⁂*^ *Candida albicans*	125	28

Kanamycin was used as a positive control for pathogens marked with (^*∗*^), Ampicillin was used for those marked with (^⁑^), and Ketoconazole was used for those marked with (^*⁂*^).

**Table 3:** GC-HRTOFMS analysis of ethyl acetate extract of *M. radiotolerans* MAMP 4754.

R.T (min:sec)	Area (%)	Molecule name	Reported biological activity	References
20:54	1.48	9-Octadecene	Antifungal, antioxidant, anticarcinogenic, and antimicrobial activity	[67, 68]
29:10	0.62	11-Tricosene		
25:11	1.12	3-Eicosene		
08:00	0.02	2,4-Dinitrophenyl acetate	Antioxidant activity, anticancer, anti-inflammatory, antibacterial, and antiviral activity	[69, 70]
20:42	2.06	Phenol, 2,5-bis(1,1-dimethylethyl)		
23:12	0.11	Phenol, 2-(1,1-dimethylethyl)-4-(1,1,3,3-tetramethylbutyl)		
18:01	0.11	Phenol, 2-(1,1-dimethylethyl)-4-methyl		
17:14	0.43	Phenol, p-tert-butyl		
14:49	3.17	Furyl hydroxymethyl ketone		
17:03	0.24	2-Acetoxy-5-hydroxyacetophenone		
13:25	13.99	2(5H)-Furanone	Anti-inflammatory, anticancer, antimicrobial, and antifungal activity	[71–73]
13:39	0.34	2-Furanmethanol, 5-methyl		
02:09	0.12	3,3′-Bifuran, 2,2′,3,3′-tetrahydro		
13:51	0.02	2,4-Dihydroxy-2,5-dimethyl-3(2H)-furan-3-one		
17:23	0.87	3,5-Diisopropoxy-1,1,1,7,7,7-hexamethyl-3,5-bis(trimethylsiloxy)tetrasiloxane	Antifungal, antitumor, antioxidant, and antimicrobial activity	[74–77]
19:40	0.83	3-Ethoxy-1,1,1,7,7,7-hexamethyl-3,5,5-tris(trimethylsiloxy)tetrasiloxane		
24:57	0.31	3-Isopropoxy-1,1,1,7,7,7-hexamethyl-3,5,5-tris(trimethylsiloxy)tetrasiloxane		

## Data Availability

The bacterial endophyte reported herein has been deposited in GenBank with the following accession number: MF133459.

## Supplementary Materials

Supplementary Materials Table 2A has been moved to the supplementary data section as it plays a crucial role in showing the difference of inhibition from the crude extract dissolved in chloroform and ethyl acetate. The table has also been referred to in the manuscript text.

## References

[1] Kumar A., Robert A. A., Kannan V. R. (2015). Exploration of endophytic microorganisms from selected medicinal plants and their control potential to multi drug resistant pathogens. *Journal of Medicinal Plants Studies*.

[2] Demain A. L., Sanchez S. (2009). Microbial drug discovery: 80 years of progress. *The Journal of Antibiotics*.

[3] Wise R. (2008). The worldwide threat of antimicrobial resistance. *Current Science*.

[4] Patridge E., Gareiss P., Kinch M. S., Hoyer D. (2016). An analysis of FDA-approved drugs: natural products and their derivatives. *Drug Discovery Today*.

[5] Davison E. K., Brimble M. A. (2019). Natural product derived privileged scaffolds in drug discovery. *Current Opinion in Chemical Biology*.

[6] Gupta P. D., Birdi T. J. (2017). Development of botanicals to combat antibiotic resistance. *Journal of Ayurveda and Integrative Medicine*.

[7] Bentley R. (2010). *Medicinal Plants*.

[8] Chen S. L., Yu H., Luo H. M., Wu Q., Li C. F., Steinmetz A. (2016). Conservation and sustainable use of medicinal plants: problems, progress, and prospects. *Chinese Medicine*.

[9] Nair D. N., Padmavathy S. (2014). Impact of endophytic microorganisms on plants, environment and humans. *The Scientific World Journal*.

[10] Atiphasaworn P., Monggoot S., Gentekaki E., Brooks S., Pripdeevech P. (2017). Antibacterial and antioxidant constituents of extracts of endophytic fungi isolated from *Ocimum basilicum* var. *thyrsiflora* leaves. *Current Microbiology*.

[11] Wang S. S. (2019). Diversity of culture-independent bacteria and antimicrobial activity of culturable endophytic bacteria isolated from different Dendrobium stems. *Scientific Reports*.

[12] Shahzad R., Khan A. L., Bilal S., Asaf S., Lee I. J. (2018). What is there in seeds? vertically transmitted endophytic resources for sustainable improvement in plant growth. *Frontiers in Plant Science*.

[13] Pandey S. S. (2018). Endophytes of withania somnifera modulate in planta content and the site of withanolide biosynthesis. *Scientific Reports*.

[14] Xu L., Zhou L., Zhao J., Li J., Li X., Wang J. (2008). Fungal endophytes from *Dioscorea zingiberensis* rhizomes and their antibacterial activity. *Letters in Applied Microbiology*.

[15] Photolo M. M., Mavumengwana V., Serepa-Dlamini M. H., Tlou M. G. (2017). Draft genome sequence of *Methylobacterium radiotolerans* strain MAMP 4754, a bacterial endophyte isolated from *Combretum erythrophyllum* in South Africa. *Genome Announcements*.

[16] Van Dien S. J., Marx C. J., O’Brien B. N., Lidstrom M. E. (2003). Genetic characterization of the carotenoid biosynthetic pathway in AM1 and isolation of a colorless mutant. *Applied and Environmental Microbiology*.

[17] Toyama H., Anthony C., Lidstrom M. E. (1998). Construction of insertion and deletion mxa mutants of *Methylobacterium extorquens* AM1 by electroporation. *FEMS Microbiology Letters*.

[18] Tani A., Sahin N., Kimbara K. (2012). *Methylobacterium oxalidis* sp. nov., isolated from leaves of *Oxalis corniculate*. *International Journal of Systematic and Evolutionary Microbiology*.

[19] Madhaiyan M., Poonguzhali S., Senthilkumar M., Lee J.-S., Lee K.-C. (2012). Methylobacterium gossipiicola sp. nov., a pink-pigmented, facultatively methylotrophic bacterium isolated from the cotton phyllosphere. *International Journal of Systematic and Evolutionary Microbiology*.

[20] Balachandran C., Duraipandiyan V., Ignacimuthu S. (2012). Cytotoxic (A549) and antimicrobial effects of *Methylobacterium* sp. isolate (ERI-135) from Nilgiris forest soil, India. *Asian Pacific Journal of Tropical Biomedicine*.

[21] Alamgir K. M., Masuda S., Fujitani Y., Fukuda F., Tani A. (2015). Production of ergothioneine by *Methylobacterium* species. *Frontiers in Microbiology*.

[22] Jasim B., Joseph A. A., John C. J., Mathew J., Radhakrishnan E. K. (2014). Isolation and characterization of plant growth promoting endophytic bacteria from the rhizome of *Zingiber officinale*. *3 Biotech*.

[23] Tsuchida T., Koga R., Shobao H., Matsumoto T., Fukatsu T. (2002). Diversity and geographic distribution of secondary endosymbiotic bacteria in natural populations of the pea aphid, *Acyrthosiphon pisum*. *Molecular Ecology*.

[24] Collins M. D., Falsen E., Brownlee K., Lawson P. A. (2004). *Helcococcus sueciensis* sp. nov., isolated from a human wound. *International Journal of Systematic and Evolutionary Microbiology*.

[25] Gupta R. M., Kale P. S., Rathi M. L., Jadhav N. N. (2015). Isolation, characterization and identification of endophytic bacteria by 16S rRNA partial sequencing technique from roots and leaves of *Prosopis cineraria* plant. *Asian Journal of Plant Science and Research*.

[26] Golding C. G., Lindsey L., Lamboo G. C., Beniac D. R., Booth T. F. (2016). The scanning electron microscope in microbiology and diagnosis of infectious disease. *Scientific Reports*.

[27] Schadler S., Burkhardt C., Kappler A. (2008). Evaluation of electron microscopic sample preparation methods and imaging techniques for characterization of cell-mineral aggregates. *Journal of Geometry*.

[28] Trease G. E., Evans W. C. (1983). *Textbook of Pharmacognosy*.

[29] Harbourne J. B. (1983). *Phytochemical Methods: A Guide to Modern Techniques of Plants Analysis*.

[30] Hoelzer K., Cummings K. J., Warnick L. D. (2011). Agar Disk diffusion and Automated Microbroth dilution produce similar antimicrobial Susceptibility testing results for Salmonella Serotypes newport, typhimurium, and 4,5,12: i-, but differ in economic cost. *Foodborne Pathogens and Disease*.

[31] Zhang H., Bai X., Wu B. (2012). Evaluation of antimicrobial activities of extracts of endophytic fungi from *Artemisia annua*. *Journal of Bangladesh Pharmacological Society*.

[32] Pessini G. L., Dias Filho B. P., Nakamura C. V., Cortez D. A. G. (2003). Antibacterial activity of extracts and neolignans from *Piper regnelli* (Miq.) C. DC. var. *pallescens* (C. DC.) Yunck. *Memórias Do Instituto Oswaldo Cruz*.

[33] Andrews J. M. (2001). Determination of minimum inhibitory concentrations. *Journal of Antimicrobial Chemotherapy*.

[34] Takao L. K., Imatomi M., Gualtieri S. C. J. (2015). Antioxidant activity and phenolic content of leaf infusions of myrtaceae species from cerrado (Brazilian savanna). *Brazilian Journal of Biology*.

[35] Green P. N., Ardley J. K. (2018). Review of the genus methylobacterium and closely related organisms: a proposal that some methylobacterium species be reclassified into a new genus, methylorubrum gen. nov. *International Journal of Systematic and Evolutionary Microbiology*.

[36] Claus D. (1992). A standardized Gram staining procedure. *World Journal of Microbiology & Biotechnology*.

[37] Gregersen T. (1978). Rapid method for distinction of Gram negative from Gram-positive bacteria. *European Journal of Applied Microbiology*.

[38] Anjum N., Chandra R. (2015). Endophytic bacteria: optimazation of isolation procedure from various medicinal plants and their preliminary characterization. *Asian Journal of Pharmaceutical and Clinical Research*.

[39] Zhao L., Xu Y., Lai X.-H., Shan C., Deng Z., Ji Y. (2015). Screening and characterization of endophytic *Bacillus* and *Paenibacillus* strains from medicinal plant Lonicera japonica for use as potential plant growth promoters. *Brazilian Journal of Microbiology*.

[40] Liu Y.-H., Guo J.-W., Salam N. (2016). Culturable endophytic bacteria associated with medicinal plant Ferula songorica: molecular phylogeny, distribution and screening for industrially important traits. *3 Biotech*.

[41] Aljuraifani A., Aldosary S., Ababutain I. (2019). In vitro antimicrobial activity of endophytes, isolated from *Moringa peregrina* growing in eastern region of Saudi Arabia. *National Academy Science Letters*.

[42] Ek-Ramos M. J., Gomez-Flores R., Orozco-Flores A. A., Rodríguez-Padilla C., González-Ochoa G., Tamez-Guerra P. (2019). Bioactive products from plant-endophytic gram-positive bacteria. *Frontiers in Microbiology*.

[43] Tejesvi M. V., Andersen B., Antcheva N. (2016). MB1533 is a defensin-like antimicrobial peptide from the intracellular meristem endophyte of Scots pine *Methylobacterium extorquens* DSM13060. *Journal of Microbial & Biochemical Technology*.

[44] Eloff J. N., Katerere D. R., Mcgaw L. J. (2008). The biological activity and chemistry of the southern African combretaceae. *Journal of Ethnopharmacology*.

[45] Masoko P. (2007). Characterization of antifungal compounds isolated from combretum and terminalia species (combretaceae).

[46] Bhatnagar S., Sahoo S., Mohapatra A. K., Behera D. R. (2012). Phytochemical analysis, antioxidant and cytotoxic activity of medicinal plant Combretum roxburghii (Family: Combretaceae). *International Journal of Drug Development and Research*.

[47] Saxena M., Saxena J., Nema R., Singh D., Gupta A. (2013). Phytochemistry of medicinal plants. *Journal of Pharmacognosy and Phytochemistry*.

[48] Kabera J. N., Semana E., Mussa A. R., He X. (2014). Plant secondary metabolites: biosynthesis, classification, function and pharmacological properties. *Journal of Pharmacy and Pharmacology*.

[49] Woolley J. G. (2001). *Encyclopedia of Life Sciences*.

[50] Lu J.-J., Bao J.-L., Chen X.-P., Huang M., Wang Y.-T. (2012). Alkaloids isolated from natural herbs as the anticancer agents. *Evidence-Based Complementary and Alternative Medicine*.

[51] Slot J. C., Rokas A. (2011). Horizontal transfer of a large and highly toxic secondary metabolic gene cluster between fungi. *Current Biology*.

[52] Strobel G. A. (2003). Endophytes as sources of bioactive products”. *Microbes and Infection*.

[53] Richards T. A., Soanes D. M., Foster P. G., Leonard G., Thornton C. R., Talbot N. J. (2009). Phylogenomic analysis demonstrates a pattern of rare and ancient horizontal gene transfer between plants and fungi. *The Plant Cell*.

[54] Gouda S., Das G., Sen S., Shin H. S., Patra J. K. (2016). Endophytes: a treasure house of bioactive compounds of medicinal importance. *Frontiers in Microbiology*.

[55] Pansanit A., Pripdeevech P. (2018). Antibacterial secondary metabolites from an endophytic fungus, arthrinium sp. MFLUCC16-1053 isolated from zingiber cassumunar. *Mycology*.

[56] Salehan N. M., Meon S., Ismail I. S. (2013). Antifungal activity of *Cosmos caudatus* extracts against seven economically important plant pathogens. *International Journal of Agricultural and Biology*.

[57] Siek T. J. (1978). Effective use of organic solvents to remove drugs from biologic specimens. *Clinical Toxicology*.

[58] Kavitha A., Prabhakar P., Vijayalakshmi M., Venkateswarlu Y. (2010). Purification and biological evaluation of the metabolites produced by *Streptomyces* sp. TK-VL_333. *Research in Microbiology*.

[59] Mtunzi F. M., Ejidike I. P., Ledwaba I. (2017). Solvent-solvent fractionations of *Combretum erythrophyllum* (Burch.) leave extract: studies of their antibacterial, antifungal, antioxidant and cytotoxicity potentials. *Asian Pacific Journal of Tropical Medicine*.

[60] Pérez-Ramírez I. F., Castaño-Tostado E., Ramírez-de León J. A., Rocha-Guzmán N. E., Reynoso-Camacho R. (2015). Effect of stevia and citric acid on the stability of phenolic compounds and in vitro antioxidant and antidiabetic capacity of a roselle (*Hibiscus sabdariffa* L.) beverage. *Food Chemistry*.

[61] Mawoza T., Ndove T. (2015). Combretum erythrophyllum (Burch.) Sond. (combretaceae): a review of its ethnomedicinal uses, phytochemistry and pharmacology. *Global Journal of Biology, Agriculture and Health Sciences*.

[62] Rogers C. B., Verotta L., Hostettman K., Chinyanganga F., Maillard M., Wolfender J.-L. (1996). Chemistry and biological properties of the African combretaceae. *Chemistry, Biological and Pharmacological Properties of African Medicinal Plants*.

[63] Eloff J. (1998). A sensitive and quick microplate method to determine the minimal inhibitory concentration of plant extracts for bacteria. *Planta Medica*.

[64] Martini N. D., Katerere D. R. P., Eloff J. N. (2004). Biological activity of five antibacterial flavonoids from *Combretum erythrophyllum* (Combretaceae). *Journal of Ethnopharmacology*.

[65] Van Vuuren S. F. (2008). Antimicrobial activity of South African medicinal plants. *Journal of Ethnopharmacology*.

[66] Martini N., Eloff J. N. (1998). The preliminary isolation of several antibacterial compounds from *Combretum erythrophyllum* (Combretaceae). *Journal of Ethnopharmacology*.

[67] Akpuaka A., Ekwenchi M., Dashak D., Dildar A. (2013). Biological activities of characterized isolates of *n*-hexane extract of *Azadirachta* Indica A. Juss(Neem) leaves. *New York Science Journal*.

[68] Godwin A., Akinpelu B., Makinde A., Aderogba M., Oyedapo O. (2015). Identification of n-hexane fraction constituents of *Archidium ohioense* (Schimp. Ex Mull) extract using GC-MS technique. *British Journal of Pharmaceutical Research*.

[69] Krishnaiah D., Devi T., Bono A., Sarbatly R. (2008). Studies on phytochemical constituents of six Malaysian medicinal plants. *Journal of Medicinal Plants Research*.

[70] Yadav R. N. S., Agarwala M. (2011). Phytochemical analysis of some medicinal plants. *Journal of Phytology*.

[71] Oskoueian E., Abdullah N., Ahmad S., Saad W. Z., Omar A. R., Ho Y. W. (2011). Bioactive compounds and biological activities of *Jatropha curcas L.* Kernel meal extract. *International Journal of Molecular Sciences*.

[72] Sharma M. D., Rautela I., Sharma N., Gahlot M., Koshy E. (2015). GC-MS analysis of phytocomponents in juice sample of Indian cane: *Saccharum Barberi*. *International Journal of Pharmaceutical Sciences and Research*.

[73] Zekeya N., Chacha M., Shahada F., Kidukuli A. (2014). Analysis of phytochemical composition of *Bersama abyssinica*by gas chromatography—mass spectrometry. *Journal of Pharmacognosy and Phytochemistry*.

[74] Keskin D., Ceyhan N., Ugur A. (2012). Chemical composition and in vitro antimicrobial activity of walnut (Juglans regia L.) green husk’s and leaves from West Anatolia. *Journal of Pure and Applied Microbiology*.

[75] Moustafa F. M., Mahmoud A. S. A., Taha T. H., Sulaiman A. A. (2013). In vitro antifungal activity of argemone ochroleuca sweet latex against some pathogenic fungi. *African Journal of Biotechnology*.

[76] Hifnawy M. S., Salam R. M. A., Rabeh M. A., Aboseada M. A. (2013). Glucosinolates, glycosidically bound volatiles and antimicrobial activity of Brassica oleraceae var. botrytis, (Soultany Cultivar). *Journal of Biology, Agriculture and Healthcare*.

[77] Joshi R., Meena R., Patni V. (2018). Comparative phytochemical analysis of bioactive constituents present in *in vitro* and *in vivo* plant parts of merremiaaegyptia and merremiadissecta. *Journal of Pharmacognosy and Phytochemistry*.

[78] Chukwujekwu J. C., van Staden J. (2016). *In vitro* antibacterial activity of *Combretum edwardsii*, *Combretum krausii*, and *Maytenus nemorosa* and their synergistic effects in combination with antibiotics. *Frontiers in Pharmacology*.

[79] Komape N., Aderogba M., Bagla V., Masoko P., Eloff J. (2014). Antibacterial and anti-oxidant activities of leaf extracts of *Combretum vendae* (Combretecacea) and the isolation of an anti-bacterial compound. *African Journal of Traditional, Complementary and Alternative Medicines*.

[80] Narkhede A., Jagtap S. (2015). Screening of amarkand species with respect to their polyphenolic content and free radical quenching potential. *International Journal of Pharma and Bio Sciences*.

[81] Prieto M. A., Rodríguez-Amado I., Vázquez J. A., Murado M. A. (2012). *β*-Carotene assay revisited. application to characterize and quantify antioxidant and prooxidant activities in a microplate. *Journal of Agricultural and Food Chemistry*.

[82] Nimse S. B., Pal D. (2015). Free radicals, natural antioxidants, and their reaction mechanisms. *Royal Society of Chemistry*.

[83] Molyneux P. (2004). The use of stable free radical diphenylpicrylhydrazyl (DPPH) for estimating antioxidant activity. *Songklanakarin Journal of Science and Technology*.

[84] Shahidi F., Yeo J. (2018). Bioactivities of phenolics by focusing on suppression of chronic diseases: a review. *International Journal of Molecular Sciences*.

[85] Cheynier V., Comte C., Davies K. M., Lattanzio V. (2013). Plant phenolics: recent advances on their biosynthesis, genetics, and ecophysiology. *Journal of Plant Physiology and Biochemistry*.

[86] Kannan M., Kumar T. S., Rao M. V. (2016). Antidiabetic and antioxidant properties of *waltheriaindica L.*, an ethnomedicinal plant. *International Journal of Pharma Research and Health Sciences*.

[87] Pawle G., Singh S. K. (2014). Antioxidant potential of endophytic fungus colletotrichum species isolated from polygala elongate. *International Journal of Pharma and Bio Sciences*.

[88] Pan F., Su T., Cai S., Wu W. (2017). Fungal endophyte-derived *Fritillaria unibracteata* var. wabuensis: diversity, antioxidant capacities in vitro and relation phenolic, flavonoid or saponin compounds. *Scientific Reports*.

[89] Martini N. D. (2002). The isolation and characterization of antibacterial compounds from combretum erythrophyllum (burch.) sond.

[90] Masoko P., Picard J., Eloff J. N. (2007). The antifungal activity of twenty-four southern African *Combretum* species (Combretaceae). *South African Journal of Botany*.

[91] Schwikkard S., Zhou B. N., Glass T. E., Sharp J. L., Mattern M. R. (2000). Bioactive compounds from *Combretum erythrophyllum*. *Journal of Natural Products*.

[92] Yogeswari S., Ramalakshmi S. N., Muthu J. M. (2012). Identification and comparative studies of different volatile fractions from monochaetia kansensis by GC-MS. *Global Journal of Pharmacology*.

[93] Naragani K., Mangamuri U., Muvva V., Poda S., Munaganti R. K. (2016). Antimicrobial potential of *Streptomyces cheonanensis* vuk-a from mangrove origin. *Journal of Pharmacy and Pharmaceutical Sciences*.

[94] Belakhdar G., Benjouad A., Abdennebi E. H. (2015). Determination of some bioactive chemical constituents from *ThesiumhumileVahl*. *Journal of Materials and Environmental Science*.

[95] Schewe T., Steffen Y., Sies H. (2008). How do dietary flavanols improve vascular function? A position paper. *Archives Biochemistry Biophysics*.

[96] Ahn E.-Y., Jiang Y., Zhang Y. (2008). Cytotoxicity of p-tyrosol and its derivatives may correlate with the inhibition of DNA replication initiation. *Oncology Reports*.

[97] Balasundram N., Sundram K., Samman S. (2006). Food chemistry phenolic compounds in plants and agri-industrial by-products: antioxidant activity, occurrence, and potential uses. *Food Chemistry*.

