# Supplementary material for: Antimicrobial and Antioxidant Properties of a Bacterial Endophyte, Methylobacterium radiotolerans MAMP 4754, Isolated from Combretum erythrophyllum Seeds
Source: Int J Microbiol. 2020 Feb 18;2020:9483670. doi: 10.1155/2020/9483670 (PMC7060864; doi:10.1155/2020/9483670)
Supplement: Supplementary Materials — Table 2A has been moved to the supplementary data section as it plays a crucial role in showing the difference of inhibition from the crude extract dissolved in chloroform and ethyl acetate. The table has also been referred to in the manuscript text. [file 9483670.f1.zip › Supplementary dataX.pdf]

Supplementary data

Table 2A. Antimicrobial activity of *M. radiotolerans* MAMP 4754 crude extract using disc diffusion method. The crude extract was resuspended in respective solvents (chloroform and ethyl acetate). Kanamycin, Ampicillin and Ketoconazole were used as positive controls (1 mg/mL). Zones of inhibition were measured in millimetres (mm). Data was reported as mean values of the crude extract tested in triplicate. Statistical analysis was performed using ANOVA and the differences were considered to be significant at $p < 0.05$

	Zone of inhibition (mm)		
**Organism**	**Chloroform extract**	**Ethyl acetate extract**	**Antibiotic control (µg/mL)**
**Bacillus subtilis*	17	18	22
**Bacillus cereus*	15	20	22
⁑*Klebsiela oxytoca*	15	24	20
⁑*Pseudomonas aeruginosa*	15	15	25
⁑*Mycobacterium smegmatis*	15	18	20
⁑*Enterococcus faecalis*	15	12	22
⁑*Escherichia coli*	15	18	24
⁂*Candida albicans*	12	17	25

Kanamycin was used as a positive control for pathogens marked with (*), Ampicillin was used for those marked with (⁑), and Ketoconazole was used for those marked with (⁂).
